# Community Member Perceptions of Dollar Stores in Baltimore City, Maryland: “They are Not Progressive for the Communities”

**DOI:** 10.1007/s40615-024-02227-2

**Published:** 2024-11-11

**Authors:** Samantha M. Sundermeir, Sydney R. Santos, Emma C. Lewis, Sara John, Julia A. Wolfson, Lisa Poirier, Shuxian Hua, Joel Gittelsohn

**Affiliations:** 1 Department of International Health, Johns Hopkins Bloomberg School of Public Health, Baltimore, MD USA; 2 The Center for Science in the Public Interest, Washington, DC USA

**Keywords:** Qualitative, Dollar store, Public policy, Retail food environment

## Abstract

Dollar stores are the fastest-growing type of food retailer in the United States, prompting policy action across the country related to their perceived negative impact on the communities they serve. However, there is little existing research that explores community member perceptions of dollar stores, which is critical to inform new, equitable policies. To address this gap in Baltimore City, Maryland, where dollar store density is high, we aimed to describe community member perceptions of dollar stores in terms of their role in the broader community. We used thematic analysis to construct themes from community member in-depth interviews (*n* = 16) and one community member workshop (*n* = 21) to understand how dollar stores are viewed in the context of the broader Baltimore City community. Six key themes were generated: (1) dollar stores contribute to neighborhood “blight,” (2) better retail is needed, (3) dollar stores meet certain community needs, (4) dollar stores do not invest enough in the community, (5) dollar stores vary in location and stock depending on race-based neighborhood qualities, and (6) product quality is low. Overall, participants acknowledged that dollar stores meet certain needs in communities in which there are few alternative retail options, but many did not view them as a benefit and desired to have other retailers instead. Participants also discussed the lack of dollar store investment in the communities they serve, and the low quality of food and non-food products offered. Future policy development should include community member perspectives to understand local context and align policies with community priorities.

## Introduction

Dollar stores (small-box discount retailers) are the fastest-growing retailers in the United States, expanding at a rate of 22% between 2000 and 2017 [1], and operate over 35,000 store locations across the country [2, 3]. Dollar stores have received negative press [4–7] in recent years for their high density in neighborhoods comprised of Black and Latine populations and census tracts with lower incomes [1, 8, 9], for violating health codes [10], and poor working conditions and treatment of employees [5]. The news media helped raise awareness of these issues, and community pushback has led over 70 municipalities to block new dollar store proposals, and over 50 municipalities to pass legislation to regulate dollar stores [11, 12]. Extant policies have included temporary pauses on granting new permits/licenses to dollar stores, limiting their density using a minimum dispersal requirement, and/or requiring new dollar stores meet certain conditions before being approved to open (e.g., requiring a community benefits agreement, requiring a certain amount of shelf or floor space be dedicated to healthy, fresh food) [11, 12].

In many cases, dollar store policy action is reportedly driven by community organizing and grassroots efforts [4, 12]. However, there is little existing research about community member perceptions of dollar stores to support evidence-based policy action. The Center for Science in the Public Interest fielded a nationwide survey in 2022 about dollar store perceptions and utilization habits [4]. They found overall positive perceptions about dollar stores, with 81% of the 750 respondents agreeing that dollar stores help their communities, as opposed to harming them [4]. In North Carolina, community members with low incomes reported relying on dollar stores because of their affordability and convenience, but wanted healthier options such as produce and high-quality proteins [13]. Given this contrast to the negative sentiment often reported in the media [4], the issue of dollar stores may be place-based, with unique challenges depending on location, and underscores the need for community-based research. Qualitative research may reveal the nuances of how dollar stores help or harm communities.

To our knowledge, just two studies have been conducted at the local level with the purpose to inform policy in DeKalb County, Georgia, and New Orleans, Louisiana [14, 15]. Both studies were mandated by policy-makers in response to community outcry for policy action, and both led to successful passage of dollar store policies in their respective locations. However, only the New Orleans study incorporated community perspectives in their design, and only via public comments submitted during a public hearing on dollar stores. Public comment on dollar stores in New Orleans demonstrated concerns about low-quality products, lack of healthy food options, poor store upkeep, insufficient staffing, crime/safety issues, and potential negative impacts on the neighborhood overall [15]. Thus, there remains a gap in the literature regarding how community members perceive dollar stores in the context of their local community.

To address this gap in Baltimore City, Maryland, where there are over 50 dollar store locations [16], we aimed to describe community member perceptions of dollar stores in terms of their role in the broader community to inform policy strategies under consideration by the City Council to regulate dollar stores [17].

## Methods

### Study Design

This is a qualitative analysis of a larger study of dollar stores in Baltimore City which employed a sequential mixed-methods approach [18] to understand the role of dollar stores in the food environment and the community. The larger study included in-depth interviews with key stakeholders, a community member workshop, a policy-maker workshop, and dollar store observations. The present study utilizes the qualitative data from interviews with community members and the community member workshop for analysis.

### Setting

This study took place in Baltimore City shortly after a period of community organizing around dollar stores to advocate for policy action [19]. In Baltimore, about two-thirds of residents identify as Black or African American, and more Black residents live in a Healthy Food Priority Area (HFPA) of the City (31%) compared to White individuals (9%) [20]. Healthy food priority areas are defined as neighborhoods where the average Healthy Food Availability Index score is 9.5/28.5 or less, the median household income is < 185% of the Federal Poverty Level, > 30% of households have no vehicle, and the distance to any supermarket is > 0.25 mi [20]. Baltimore has a long history of racial segregation and discriminatory investment practices which created the “Black Butterfly” (the modern-day shape of the city’s racial makeup created by historically segregated Black neighborhoods) where there are higher rates of poverty and less access to important resources such as healthy food [20, 21]. Given this history, is it critical to include community voices in policy-making to ensure dollar store policies are effective and equitable to those they impact the most [22].

### Inclusion Criteria

To be eligible to participate in interviews or the workshop, participants had to be at least 18 years old and currently live in Baltimore City. For the community member workshop, our recruitment prioritized participants who expressed interest in the workshop and who shopped at dollar stores at least once per month in order to contribute to the discussion based on their experiences at dollar stores. Two workshop participants lived in Baltimore County, but worked in Baltimore City and were allowed to participate given their extensive knowledge of Baltimore and the communities they work in.

### In-depth Interviews

In-depth, semi-structured interviews were conducted with Baltimore City residents from December 2022 to June 2023. While we did not directly collect information on occupation, three of the participants shared that they either worked at a dollar store (*n* = 1) or another food retailer (*n* = 1), or owned a local business (*n* = 1) which may provide additional perspective. Interview guides were developed iteratively through discussion among the research team and project partners. Interview guides probed on the food retail landscape in one’s neighborhood overall, perceptions of or experiences with how dollar stores are used to meet shopping needs, what types of products are purchased there, and what an “ideal” dollar store might look like. Data collectors (SMS, SRS) were trained graduate student research assistants from the Johns Hopkins Bloomberg School of Public Health who completed qualitative methods coursework and had experience conducting interviews. Participants completed one 45–60-min interview via Zoom (*n* = 14) or in-person (*n* = 2) depending on their preference, and were asked open-ended questions about their views on dollar stores in their neighborhoods. Participants were aware that the interviewers were graduate students at the Johns Hopkins Bloomberg School of Public Health but had no prior relationship. The interviews were recorded and transcribed using the transcription feature in Microsoft Word, and cleaned and checked for accuracy by research assistants. The research team met weekly to discuss emerging topics and data saturation.

### Community Workshop

The Baltimore City community member workshop occurred in July, 2023. Although the primary purpose of the workshop was to co-generate dollar store policy recommendations [17], discussions regarding the role of dollar stores in the community and how people view them in Baltimore City occurred as well. Workshop guides were developed by the research team and policy staff from the Center for Science in the Public Interest (CSPI). The workshops were led by faculty and students at the Johns Hopkins Bloomberg School of Public Health, and two policy staff from CSPI, and were introduced as such at the start of the workshop. One researcher had extensive experience facilitating workshops (JG). The workshop was recorded using digital audio recorders, and were transcribed verbatim by research assistants. Two of the interviewees from the previous phase also participated in the workshop. Field notes were discussed and recorded at the end of the workshop.

The community member workshop was held in a public meeting space in Baltimore City, and was divided into two parts with a 10-min break halfway through. To start, each participant introduced themselves by stating their name, neighborhood, and one asset and one challenge in their neighborhood. Key words were recorded on a flipchart. Then, participants were allocated ten votes (sticky dots) to vote for the most important challenges among those generated. The votes were tallied and the top three challenges were announced. Next, the focus transitioned to dollar stores by asking participants how dollar stores fit into the previous discussion about neighborhood strengths and challenges, and the top three challenges identified based on their votes. Key words/phrases in response to this were recorded on the flipchart. To finish the first half of the workshop, a short PowerPoint about the dollar store study findings was presented, and closed by putting the top five policy options generated from the larger study up on the screen. The second half of the workshop focused on generating policy recommendations and has been published elsewhere [16]. The workshop lasted three hours and 15 min.

### Recruitment

For interviews, participants were recruited using purposive, snowball, and convenience sampling. Purposive sampling was used to recruit community members who were previously involved in dollar store issues and policy in Baltimore City suggested by the State Senator who introduced Senate Bill 869. Snowball sampling was used thereafter by asking interviewees who else should be interviewed, and inviting them to share the study flyer with their friends and family. For both interview and workshop recruitment, the research team attended neighborhood association meetings, and distributed/posted flyers at community locations (retail stores, libraries, recreation centers, and community events) and via email listservs including those managed by neighborhood associations, City Council offices, and the Food Policy Action Coalition. For those who were not already familiar with dollar store issues in Baltimore, participants were told the study was focused on understanding how people view and utilize dollar stores in Baltimore City.

Sixteen community members of the 27 that responded to interview recruitment efforts declined to participate due to time conflicts or were excluded if from communities that were already overrepresented among interviewees (no more than three to four per neighborhood). Fifty community members expressed interest in attending the community workshop, and 33 were ultimately invited based on meeting age and residence requirements, and prioritizing those who reported shopping at dollar stores and/or lived in underrepresented zip codes. Of those invited, 21 ultimately attended. Workshop participants completed a paper demographic survey that assessed age, race/ethnicity, sex, zip code, food security status, and dollar store shopping frequency.

### Ethical Considerations

This study was approved by the Johns Hopkins School of Public Health Institutional Review Board (IRB00022523). All participants completed informed consent prior to engaging in an interview or the workshop. Participants received electronic gift cards for completing and interview, and physical gift cards for completing the workshop as compensation for their time.

### Analysis

An inductive coding approach and thematic analysis were used to analyze the transcripts, rooted in grounded theory [23, 24]. We used a six-stage thematic analysis process (data familiarization, generating initial codes, generating initial themes, reviewing themes, defining and naming themes, and writing up [25]. An initial set of codes were developed based on data familiarization and interview debriefing among the research team. Two coders (SMS, SRS) coded the first four transcripts and reconvened to discuss and update the codebook by adjusting code definitions and generating new codes to be applied. Once the codebook was finalized, the first author (SMS) completed coding for the remaining transcripts. Thematic analysis was used to construct initial themes by grouping codes into larger topics. The themes were reviewed and discussed iteratively during weekly team meetings until the themes, the name of each theme, and their definitions were finalized. All coding was completed using Dedoose version 9 [26].

## Results

At total of 35 unique community members participated in either an in-depth interview and/or attended the workshop. Six of the 13 (46%) interview participants reported living in a HFPA. Twenty-one Baltimore residents participated in the community member workshop. The majority of participants (63%) identified as Black, and 44% reported experiencing food insecurity in the last 12 months. See Table 1.

**Table 1:** Characteristics of 35^*^ interviewees and workshop participants in a study of dollar stores in Baltimore City, Maryland, USA

Study phase	*N*	Characteristic	*N* (%)
In-depth interviews	16	% Living in a healthy food priority area	7 (43.8)
Community member workshop	19^†^	% Living in a healthy food priority area	10(52.6)
		Race/ethnicity	
		Asian	1 (5.3)
		Black	12 (63.2)
		White	5 (26.3)
		Declined to answer	1 (5.3)
		Age	
		18–24	0
		25–34	3 (15.8)
		35–44	3 (15.8)
		45–54	2 10.5)
		55–64	7 (36.8)
		65 +	4 (21.1)
		% Female	14 (73.7)
		Educational attainment	
		Some high school	1 (5.3)
		High school graduate	3 (15.8)
		Vocational/two-year/community college	3 (15.8)
		Some 4-year college	1 (5.3)
		Four-year college graduate	2 (10.5)
		Advanced degree	9 (47.4)
		Experiencing food insecurity (% Yes) (*n* = 18)	8 (44.4)
		Dollar store shopping frequency	0
		Daily	3 (15.8)
		A few times/week	2 (10.5)
		Once per week	7 (36.8)
		A few times/month	2 (10.5)
		Never	

*Two participants completed an interview and the workshop, however their data from the workshop was anonymous and therefore cannot be separated out. Therefore, the data for those two participants is presented in both rows

^†^There were 21 workshop participants. Two of them declined to complete the questionnaire leaving a total *N* of 19 for demographic analysis

Six overarching themes were constructed from the data: (1) dollar stores contribute to neighborhood “blight,” (2) better retail is needed, (3) dollar stores vary in location and stock depending on race-based neighborhood qualities (4) dollar stores do not invest enough in the community, (5) product quality is low, and (6) dollar stores meet community needs.

### Dollar Stores Contribute to Neighborhood “Blight”

There was an over-arching sentiment that dollar stores, at least in their current state, are a “blight” to the neighborhoods where they are located. Within this theme, three sub-themes emerged that help to explain why community members feel this way: (1) lack of store upkeep, (2) health hazards, and (3) poor security and high crime.

Lack of dollar store upkeep was highly emphasized by most participants. They discussed increased trash surrounding the stores, and lack of cleanliness, clutter, and disarray inside the stores. Overall, participants did not think dollar stores did a sufficient job at maintaining their stores and surrounding property, leading many stores to become an eye-sore in the community and failing to live up to the quality and standards for retailers they would like to see in their neighborhoods:

*“...my main issue has been that for every dollar store that we have on the West side of Baltimore, it’s a dirty store. Dirty inside, and dirty outside. That’s it. If you want to be in the neighborhood, help clean up the neighborhood!”* – Community Member

Concerns about health hazards stemmed from lack of store upkeep as well as the lack of healthy food availability at dollar stores. Given a number of recent Occupational Safety and Health Administration (OSHA) violations at dollar stores across the country [10], participants view cleanliness issues, store clutter and disorganization, and chronic understaffing to be health hazards to dollar store employees and customers, and thus a further detriment to the neighborhood overall:

*“[The previous] OSHA comment is because there are employees who work there are fined for emergency exits being blocked. The lady who is blind, she can't get down the aisle. That's a health hazard. The employees that work there, we have all gone to one of these stores with just one person [working] and that person is missing. All of those are hazards, those are health hazards.”* – Community Member

Participants also noticed the lack of healthy food options and high availability of high-sugar, high-fat, and high-sodium foods available at dollar stores. They emphasized the importance of this given that some community members do not have other options, such as a grocery store, nearby, lack transportation to access other food retailers, and have limited budgets. Participants discussed how this contributes to the overall health and wellbeing of the members of their communities:

*“So I think it's like having these chain dollar stores, in what we would call Healthy Food Priority Areas, as like the de facto, maybe walkable to your house, place to shop is not what residents want.”* – Community Member

Many discussed the role dollar stores may play in security and crime related issues. Several participants reporting seeing or hearing about high rates of theft at dollar stores, which they attribute to stores being understaffed and cluttered, making it easier to steal products. Given the high rates of theft, participants speculated that this may exacerbate difficulties finding staff for the store, and, this was tied back to the overall health and wellbeing of the dollar store employees who are often from the surrounding community. They mentioned that these issues bring unwanted activity to the area, increasing community members’ concerns about neighborhood safety:

*“So there's people who hang on that side street, the back of the [dollar] store is on a side street. So you see people hanging back there. The police have had to be called numerous times already because of the loitering, the people hanging out there. Of course there's drugs, but you know, it just gives police more work to do unnecessarily.”* – Community Member

### Better Retail is Needed

There was a strong desire for better retail among participants, and they felt that dollar stores “*are not progressive for the communities*” (Community Member) because they (1) do not meet the standards for retail they would like to see in their neighborhoods, and (2) may prevent other, better retailers from opening. Participants want more retail options that would be beneficial to the neighborhood and/or fill a need that the neighborhood wants filled. Throughout the interview process, participants mentioned that many Baltimore City neighborhoods have some version of a strategic plan or broader vision for their neighborhood which outlines specific plans, including for retailers, which they hope to accomplish in future years. Some felt that dollar stores are not progressive for their neighborhoods and do not align with the vision they are working towards to improve the area:

*“And then [dollar stores] block out other kinds of options that we could have, and nicer places that would be beneficial to the neighborhood and supported by the neighborhood.”* – Community Member

Participants preferred to have a greater diversity of retail options such as coffee shops, restaurants, and clothing stores as such retailers would be beneficial to the neighborhood and attract people to the area.

### Dollar Stores Vary in Location and Stock Depending on Race-based Neighborhood Qualities

Some participants noted a higher density of dollar stores depending on the location, including comparisons among non-White and White neighborhoods, and city versus county (Baltimore County has a higher proportion of White residents compared to Baltimore City [27]):

*“They don't have them in a lot of White communities. They have them in more African-American communities, I'll say Brown communities, because you don't see them in Ellicott City. You don't see them once you go past a certain point, like Howard County.”* – Community Member

There was also a perception that dollar stores in White communities have higher quality stores and products:

*“So my issue when it comes to Dollar Tree and Dollar General and the Family Dollars is that when I go into the Caucasian neighborhoods like Canton or Upper Fells, it’s lit, the aisles are clean, the shelves are stocked. But when I go into the one on Greenmount, it’s dark, the staff is nasty, and so forth and so on.”* – Community Member

Some discussed that discrepancies are rooted in historical issues like systemic racism, specifically racially restrictive community covenants and zoning ordinances in Baltimore City, which have created the Black Butterfly [21]. Community members felt that the presence of dollar stores in neighborhoods today are perpetuating such intergenerational issues:

*“And if you talk about the Black Butterfly, that is where all of the dollar stores are. And that brings down the real estate value in that neighborhood, so it becomes it becomes a self-fulfilling prophecy that the neighborhood can’t change.”* – Community Member

### Dollar Stores Do Not Invest Enough in the Community

Given the sheer number of dollar stores within Baltimore City limits, participants felt that dollar stores should do more to invest back into the communities they operate in, for example, by hiring local staff, participating in local fresh produce programs, and helping keep the neighborhood clean. There was also a desire for dollar stores to be more involved in the community, similar to other businesses, who keep up with landscaping and participate in community events:

*“I mean McDonald’s is a great example, they sometimes come and do landscaping around there. There’s another fast-food restaurant that sits on our board meetings. They can, you do have to reach out to corporate, but [dollar stores] should be investing more in the local community...”* – Community Member

### Product Quality is Low

The food and non-food products sold at dollar stores are perceived as cheap and low in quality. In terms of food, participants described this as a lack of healthy food availability. In terms of non-food products, participants viewed products as being cheap and quick to break. There was a sense of “you get what you pay for” in the discussion, which results in low expectations for what types of products you can get at the dollar stores. But, at the same time, participants recognized that some community members do not have another shopping option. Similarly, some participants were not necessarily surprised by a retailer selling low-quality products as they are unfortunately used to experiencing this:

*“Because [dollar stores are] geared towards poor people, even the stuff that they sell is cheaply made because they know “OK, we're gonna send it here because this is the only thing they can afford, and they're gonna buy it anyway”. So, you know, they send it there.”* – Community Member

### Dollar Stores Meet Certain Community Needs

In contrast, participants acknowledged that dollar stores meet a need in the communities in which they are located. This comes from an understanding among participants that some community members do not have access to transportation and rely on nearby stores to meet their shopping needs. Participants liked that dollar stores stock some staple food items like dairy, and canned and frozen fruits and vegetables. They also provide other household items at affordable prices, and some specialty items that you may not find elsewhere:

*“But if they were to leave the neighborhood, I think it would be a loss. I think it would be a loss because there's so many things that you can get there that you can't get in the mall. You can buy tennis shoes and jeans and pants and shirts and all of that stuff. But if you have a class of children or adults and you want to do arts and crafts, you need items from the dollar store. Either the Dollar Tree store or the Family Dollar. These stores will help you with that aspect of your improvements to some of your projects or activities for the individuals in the area.”* – Community Member

These considerations led participants to prefer finding ways to improve the existing dollar stores in their neighborhoods to better serve those who rely on them.

## Discussion

In this qualitative study of community member perspectives on dollar stores in Baltimore City, participants generally acknowledged that dollar stores meet certain community needs, but did not view them as a benefit and desired to have other retailers instead. A prominent theme was that dollar stores are a “blight” on the neighborhoods they operate in given the unsightly and unclean store appearance, their perceived impacts on the health of dollar store staff and customers, and their contribution to increased crime and security concerns in the surrounding area. The findings from this study were reported to the Baltimore City Council via a written report. City Council introduced a conditional use dollar store bill in October of 2023 (Council Bill 23–0431), which would require new dollar stores to be at least ½ mile from existing dollar stores. The Bill is currently under review.

The findings align with public comments about dollar stores received by the New Orleans planning commission; their findings highlighted concerns about low-quality products, high unhealthy and processed food availability, poor store upkeep, insufficient staffing, crime/safety, and impact on the neighborhood overall including property values and likelihood of grocery store entry [15]. The discussion around dollar stores and their role in attracting and/or perpetuating crime was not surprising given recent news reporting on theft and violence at dollar stores [5, 28, 29]. This finding also aligns with the dollar store study conducted in DeKalb County, Georgia, which found higher levels of crime activity within 100 feet of dollar stores, and that the number of dollar stores in a given area was significantly positively associated with the total number of crimes in that area [14].

Participants described dollar stores as a symptom or indicator of historical and present-day structural racism, retailer redlining, and disinvestment [8, 30]. Retailers, especially large format stores like supermarkets, ultimately play a role in shaping structural issues such as inequitable food access by operating in particular neighborhoods based on sociodemographic characteristics including race and income [8]. In certain areas, this may leave a gap in the retail market that can and have been filled with dollar stores, particularly in Baltimore City where most of the dollar stores are located in socially vulnerable neighborhoods [8, 31, 32]. In terms of disinvestment, a recent study by the Urban Health Institute found that capital investments are distributed unevenly across Baltimore City, driven by factors such as poverty and race; majority-White neighborhoods received more than three times the investments that majority-Black neighborhoods received between 2004 and 2016 [30].

The conversation around how dollar stores meet certain needs in the community echoes the overall positive sentiment about dollar stores described in CSPI’s nationwide survey [4]. Therefore, two important policy implications can be drawn from this work. First, future policy development should explore ways to improve existing dollar stores in Baltimore City, in addition to current efforts to limit their proliferation. Dollar store policies passed in other locations and the dollar store bill recently introduced in Baltimore City apply only to new dollar stores opening in a specific area. Thus, they do not address concerns raised about existing stores such as appearance and cleanliness, health, security and crime, and staff treatment. Second, participants in this study had a strong desire for other retailers beyond dollar stores to help improve their neighborhoods. More policies that successfully incentivize or attract retailers that the community wants may help neighborhoods uphold their strategic plan/vision, give them a say in who operates in their neighborhood, and ensure retailers are serving the needs of the communities in which they are located.

This study is limited to a sample of 35 Baltimore residents, and may not be representative of how all community members view dollar stores. However, the data is nested in a larger mixed-methods study of dollar stores in Baltimore City which provided additional context. Further analysis by participant neighborhood HFPA status may have been useful, but was not possible due to our inability to accurately identify individual workshop participants in the audio recording. The research team is comprised largely of White female researchers, and about half live in Baltimore City. Five members of the research team hold PhDs, and three hold master’s degrees. We acknowledge the biases that this positionality may bring to the research. Community members and partners helped guide this research including providing input on who to contact for interviews and workshop participation, what types of questions they wanted answered through this work, and interpretation of findings through the community member workshop which was the culmination of the larger study.

## Conclusion

Participants acknowledged that dollar stores meet certain community needs, but did not view them as a benefit and desired to have other retailers instead. Community members would like the existing dollar stores to contribute to the communities they serve by creating cleaner, healthier stores and investing in the surrounding neighborhood. Future policy development should include community member perspectives to understand local context, and explore ways to improve existing dollar stores in Baltimore City and incentivize or attract retailers desired by the community.

## Data Availability

The data can be made available upon request.
